# Border Terriers under primary veterinary care in England: demography and disorders

**DOI:** 10.1186/s40575-017-0055-3

**Published:** 2017-11-25

**Authors:** Dan G. O’Neill, Elisabeth C. Darwent, David B. Church, Dave C. Brodbelt

**Affiliations:** 1 Pathobiology and Population Sciences, The Royal Veterinary College, Hawkshead Lane, North Mymms, Hatfield, Herts AL9 7TA UK; 2 The Royal Veterinary College, Hawkshead Lane, North Mymms, Hatfield, Herts AL9 7TA UK; 3 Clinical Sciences and Services, The Royal Veterinary College, Hawkshead Lane, North Mymms, Hatfield, Herts AL9 7TA UK

**Keywords:** VetCompass, Electronic patient record, Breed, Dog, Epidemiology, Prevalence, Pedigree, Crossbred, Syndromic

## Abstract

**Background:**

The Border Terrier is a working terrier type that is generally considered to be a relatively healthy and hardy breed. This study aimed to characterise the demography and common disorders of Border Terriers receiving veterinary care in England using de-identified electronic patient record data within the VetCompass™ Programme.

**Results:**

Annual birth proportion for Border Terriers showed a decreasing trend from 1.46% in 2005 to 0.78% in 2014. The median adult bodyweight for males (10.9 kg, IQR: 9.6–12.3, range: 6.3–25.0) was higher than for females (9.1 kg, IQR: 8.2–10.3, range: 5.2–21.6) (*P* < 0.001). The median longevity was 12.7 years (IQR 9.3–14.3, range 1.0–17.5).

The most prevalent fine-level disorders recorded were periodontal disease (17.63%, 95% CI: 15.62–19.79), overweight/obesity (7.01%, 95% CI: 5.69–8.52) and otitis externa (6.71%, 95% CI: 5.42–8.19). The most prevalent grouped-level precision disorders were dental disorder (18.54%, 95% CI: 16.48–20.74), enteropathy (11.68%, 95% CI: 10.00–13.53), and skin disorder (10.17%, 95% CI: 8.60–11.93).

Syndromic analysis showed that the most prevalent body locations affected were the head-and-neck (37.75%, 95% CI: 35.14–40.43), abdomen (18.61%, 95% CI: 16.55–20.81) and limb (11.53%, 95% CI: 9.86–13.37). At least one organ system was affected in 834 (62.85%) Border Terriers. The most prevalent organ systems affected were the digestive (32.03%, 95% CI: 29.52–34.61), integument (26.68%, 95% CI: 24.31–29.14), connective/soft tissue (11.15%, 95% CI: 9.51–12.97) and auditory (9.87%, 95% CI: 8.32–11.60). At least one affected pathophysiological process was described in 881 (66.39%) Border Terriers. The most prevalent pathophysiologic processes recorded were inflammation (31.65%, 95% CI: 29.15–34.23), nutritional (9.04%, 95% CI: 7.55–10.72), mass/swelling (8.89%, 95% CI: 7.42–10.55), traumatic (7.99%, 95% CI: 6.59–9.58) and infectious (7.76%, 95% CI: 6.38–9.33).

**Conclusions:**

This study documented a trend towards reducing ownership and relatively long-livedness in the Border Terrier. The most common disorders were periodontal disease, overweight/obesity and otitis externa. Predisposition to dental and neurological disease was suggested. These results can provide a comprehensive evidence resource to support breed-based health plans that can contribute positively to reforms to improve health and welfare within the breed.

## Plain English summary

The Border Terrier has been reported to be predisposed to a range of conditions. However, predisposition does not necessarily mean that a disease is common or even important for the breed. To know this, additional information on, amongst other things, prevalence, is required. This study aimed to describe the breed demography and the frequency of the most common disorders of the general population of Border Terriers in England. The study used veterinary clinical information collected within the VetCompass™ Programme at the Royal Veterinary College. Information available included demographic (breed, age, sex, neuter, colour, insurance and bodyweight) and clinical information.

Border Terriers comprised 2841 (1.08%) of the overall 264,260 study dogs but showed a decreasing trend from over 1.4% of all puppies born in 2005 to less than 0.8% of puppies born in 2014. The average bodyweight males (10.9 kg) was higher than females (9.1 kg).

The average lifespan was 12.7 years.

Of 1327 Border Terriers assessed that were under veterinary care during 2013, 881 (66.4%) had at least one disorder recorded during 2013. The most common disorders recorded were dental disease (17.63% of dogs) overweight/obesity (7.01%), ear infections (6.71%), overlong nails (5.28%), anal sac impaction (4.75%), and vomiting (4.37%).

This study documented a trend towards reducing ownership in England. The Border Terrier is shown to be a relatively long-lived breed. Specific predisposition to dental and neurological disease were suggested. These results can provide a useful evidence resource to support breed-based health plans for the Border Terrier.

## Background

The borders area of Northumberland and Scotland gave its name to the Border Terrier around 1880 when the breed was originally was used as an earth dog to support the work of the Border Foxhounds [1]. The Kennel Club (KC) Breed Standard which describes the ideal characteristics, temperament and appearance of pedigreed dogs states that absolute soundness is critical for the breed which should essentially be a working terrier and obvious conditions or exaggerations which would be detrimental in any way should be avoided [2]. Considered by the KC to be a relatively healthy and hardy breed, no specific veterinary screening schemes or DNA tests for disease are mandated for the Border Terrier under the KC’s Assured Breeder Scheme [1]. However, despite apparent health, Kennel Club registrations for the Border Terrier have declined recently in the UK. The breed dropped from 8th most popular breed in 2007 with 8814 registrations (3.3% of all registrations) to 10th position in 2016 with 5150 registrations (2.3% of all registrations) although data on breed numbers in the wider general population are scant [3].

The Border Terrier has been reported as predisposed to generalised sebaceous gland hyperplasia [4], canine epileptoid cramping syndrome [5], epilepsy [6], corneal ulceration [7], ectopic ureter [8] and dystocia [9]. However, although predisposition implies a higher predilection for a specific disorder compared with some other comparator group, it does not provide strong evidence of a substantial welfare impact per se. To better understand the welfare relevance of a disorder to a breed, additional information on prevalence, severity and duration for individual disorders are required [10]. Some efforts have been made to estimate and rank the welfare impact of various disorders in dogs using a scoring system called the Generic Illness Severity Index for Dogs (GISID) [11, 12] but these attempts have been largely frustrated by a well-recognised deficiency of reliable, up to date and generalisable prevalence data [13]. Few purpose-designed prevalence studies have been reported concerning either the overall or breed-specific canine populations [12].

The aims of this current purpose-designed prevalence study were to characterise the demography and common disorders of the general population of Border Terriers receiving veterinary care in England in order to augment the extant evidence base on health for the breed. Specific objectives were to describe the demographic composition including annual birth proportion, age distributions and bodyweight growth patterns, to estimate the prevalence of common disorders recorded in Border Terriers and to report on the most common body locations, organ systems and pathophysiological processes affected. The results from the current study could therefore assist owners and veterinarians with awareness of key conditions in the breed and to provide a reliable framework for future reforms in breeding practices that can ultimately contribute to improved health and welfare of Border Terriers.

## Methods

The VetCompass™ Programme at the Royal Veterinary College collates de-identified electronic patient record (EPR) data from primary-care veterinary practices in the UK for epidemiological research [14, 15]. The methods used for general prevalence studies have been described in detail previously [15]. Briefly, collaborating practices were selected by their willingness to participate and their recording of clinical data within an appropriately configured practice management system. Practitioners could record summary diagnosis terms from an embedded VeNom Code list during episodes of care [16]. Information collected related mainly to the owned dog population and included patient demographic (species, breed, date of birth, sex, neuter status, colour, insurance status and bodyweight) and clinical information (free-form text clinical notes, summary diagnosis terms and treatment, with relevant dates) data fields.

The sampling frame for the current study included all dogs with at least one EPR (clinical note, VeNom summary diagnosis term, bodyweight or treatment) recorded in the VetCompass™ database during the study period from September 1st, 2009 to March 9th, 2015. These dogs were accepted as being *under veterinary care during the study period* and this census of all dogs available in the database comprised the denominator group in the demographic sections of the study. Date data associated with each EPR event (clinical note, VeNom summary diagnosis term, bodyweight or treatment) were extracted and those dogs with a) at least one EPR recorded during 2013 or b) at least one EPR recorded both before and after 2013 were accepted as being *under veterinary care during 2013* and were included as the denominator group for the disorder prevalence sections of the study in order to report one year period prevalence values. Ethics approval was granted by the RVC Ethics and Welfare Committee (reference number 2015/T310).

### Demography

Demographic evaluation used data recorded on all dogs in the VetCompass™ database *under veterinary care during the study period.* Dogs recorded as Border Terrier breed were categorised as Border Terrier and all remaining dogs were categorised as non-Border Terrier. Using the birth dates recorded in the EPRs, the year of birth was derived for all dogs born from 2005 to 2014. Annual birth proportion for Border Terriers described the proportion of Border Terriers relative to all dogs born in each year from 2005 to 2014 and were reported using percentage values. All-age bodyweight data with their associated dates were used to generate bodyweight growth curves for male and female Border Terriers by plotting age-specific bodyweights and were overlaid with a cross medians line plot using the Stata *mband* command. Coat colour data recorded in the EPRs were used to describe the most common colors of Border Terriers.

### Disorder prevalence

Disorder prevalence evaluation used clinical data recorded on Border Terriers in the VetCompass™ database that were *under veterinary care during 2013* in order to report one-year period prevalence values. *Age* described the age (years) for each Border Terrier at the earlier of either December 31st, 2013 or the date of death and was categorised into six groups (< 3.0, 3.0–5.9, 6.0–8.9, 9.0–11.9, ≥ 12.0, not recorded). *Bodyweight* described the maximum bodyweight recorded during the study period for mature dogs (older than nine months) and was categorised into eight groups (0.0–4.9 kg, 5.0–6.9 kg, 7.0–8.9 kg, 9.0–10.9 kg, 11.0–12.9 kg, 13.0–14.9 kg, ≥ 15.0 kg, not recorded). N*euter* described the status of the dog (neutered or entire) recorded at the final EPR and i*nsurance* described whether a dog was insured at any point during the study period.

All clinical notes and VeNom summary diagnosis terms recorded from January 1st, 2013 to December 31st, 2013 for Border Terriers under veterinary care during 2013 were reviewed in detail and the most definitive diagnostic term recorded for each disorder was manually linked to the most appropriate VeNom term as previously described [15]. Elective (e.g. neutering) or prophylactic (e.g. vaccination) clinical events were not included. Multiple counting of disorder events for ongoing cases was avoided by including recurring diagnoses of ongoing conditions only once (e.g. repeated events of otitis externa) and by including only the final diagnosis for cases with diagnostic refinement over time (e.g. following clinical work-up or trial therapy), based on the assumption that diagnostic accuracy increased over time [17]. The parent term was used for disorders that encompassed multiple child terms [18] (e.g. a parent term *traumatic injury* may have multiple child terms such as *laceration*, *fracture* and *hypovolaemic shock*). Disorder events that were aetiologically independent despite sharing the same disorder term name (e.g. novel traumatic events) were included separately. No distinction was made between pre-existing and incident disorder presentations. Disorders described within the clinical notes using presenting sign terms (e.g. ‘vomiting’ or ‘vomiting and diarrhoea’), but without a formal clinical diagnostic term being recorded, were included using the first sign listed for each disorder (e.g. vomiting). Dental disorders were included only where at least one clinical management intervention was recommended.

The extracted VeNom diagnosis terms were mapped to three interpretation systems for analysis: fine-level precision, grouped-level precision and syndromic classification as previously described [15]. In brief, fine-level precision terms were one-to-one descriptors of the original extracted terms describing the maximal diagnostic precision recorded within the clinical notes (e.g. *inflammatory bowel disease* would remain as *inflammatory bowel disease*). Grouped-level precision terms were one-to-one descriptors of original diagnosis terms mapped to a general level of diagnostic precision (e.g. *inflammatory bowel disease* would map to *enteropathy*). Syndromic classification mapped the original VeNom diagnosis terms to three taxonomic groupings: body location, organ system and pathophysiologic process, and each original diagnostic term could be mapped to more than one syndromic terms [15].

Following data checking and cleaning in Excel (Microsoft Office Excel 2013, Microsoft Corp.), analyses were conducted using Stata Version 13 (Stata Corporation). The sex, neuter status, insurance, age and adult bodyweight for Border Terriers under veterinary care during 2013 were described. A cross-sectional study design was used to estimate one-year (2013) period prevalence with 95% confidence intervals (CI) that described the probability for disorders occurring at least once during 2013. The CI estimates were derived from standard errors based on approximation to the normal distribution for disorders with ten or more events [19] or the Wilson approximation method for disorders with fewer than ten events [20]. These methods were applied to describe prevalence based on fine-level precision, grouped-level precision and syndromic classification.

## Results

### Demography – Border Terriers during the entire study period

**Fig. 1:**
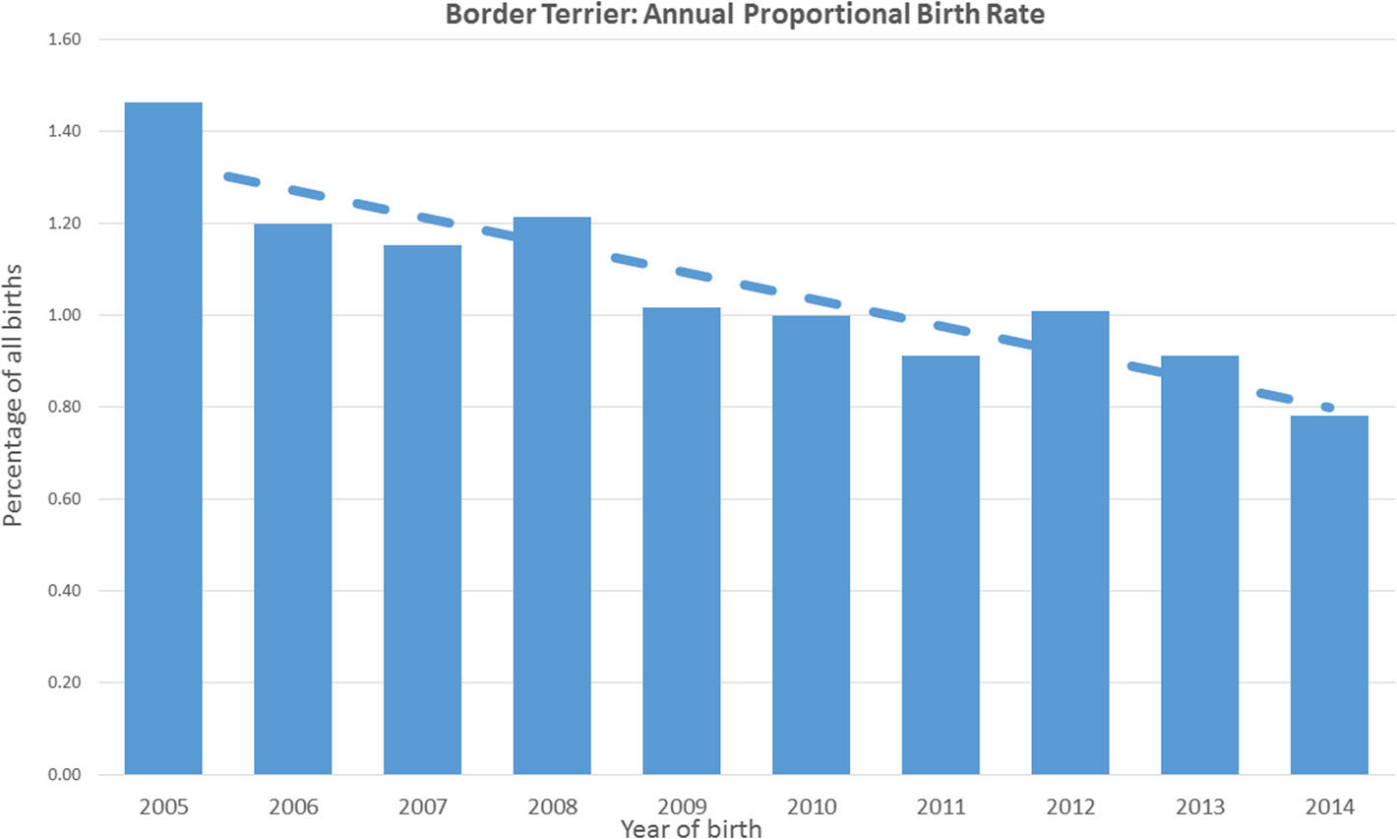
Annual birth proportion (2005–2014) for Border Terriers among all dogs under primary veterinary care at clinics in England within the VetCompass™ Programme (*n* = 263,456)

The overall sampling frame used as the denominator group for demographic analysis included 264,260 dogs at 127 clinics *under veterinary care during the study period* from September 1st, 2009 to March 9th, 2015, of which there were 2841 (1.08%) Border Terriers. Of the 263,456 (99.70% of the sampling frame) dogs with a valid date of birth available, there were 2833 (1.08%) Border Terriers. Annual birth proportion for Border Terriers dropped from 1.46% in 2005 to 0.78% in 2014 (Fig. 1).

**Fig. 2:**
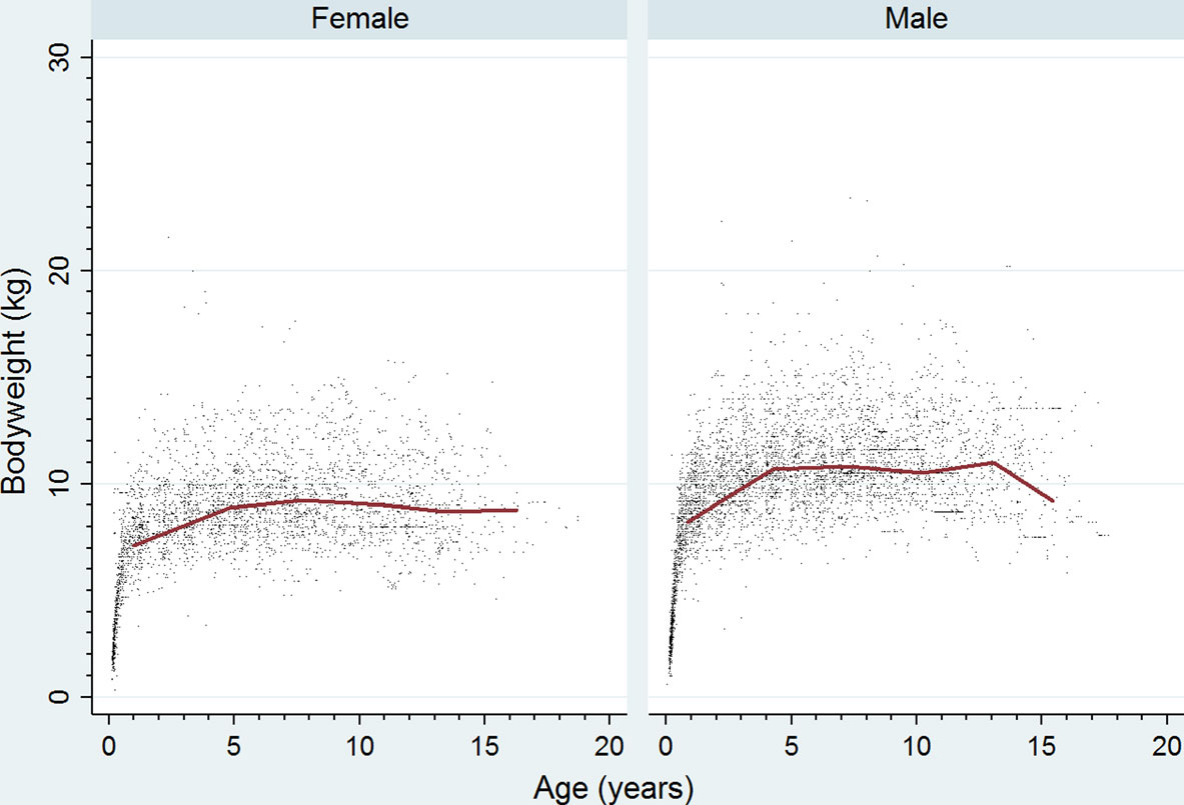
Bodyweight growth curves (kg) overlaid with a cross medians line plot for 908 female and 1180 male Border Terriers under primary veterinary care at clinics in England participating in the VetCompass™ Programme

There were 5474 unique bodyweight-date values available from 1180 male Border Terriers that had at least one bodyweight value recorded and 3901 bodyweight-date values available from 908 female Border Terriers that had at least one bodyweight value recorded. Bodyweight growth curves showed that Border Terriers puppies grow rapidly during their first nine months before entering a slower weight gain phase up to 5 years that is followed by a flatter weight plateau thereafter (Fig. 2).

Coat colour data were available for 2798 of the 2841 Border Terriers (98.49%). The most common colours recorded overall were red (*n* = 993, 35.0%), grizzle (678, 23.9%), wheaten (513, 18.1%), black (347, 12.2%) and blue (255, 9.0%). Sex data were available on 2830 dogs of the 2841 Border Terriers, (99.6%), of which 1253 (44.3%) were female and 1577 (55.7%) were male.

### Demography and common disorders for Border Terriers during 2013

There were 104,233 dogs *under veterinary care during 2013* that had either at least one EPR recorded from January 1st 2013 to December 31st 2013 (96,319 dogs) or otherwise at least one EPR before and after 2013 (7914 dogs). Border Terriers comprised 1327 (1.27%) of these dogs and were used as the denominator group for disorder prevalence evaluation. The 1327 Border Terriers were registered at 102 veterinary practices, with a median count of 20 Border Terriers per practice (interquartile range [IQR] 12–28, range 1–46). There were 881 (66.4%) Border Terriers with at least one disorder recorded during 2013 while the remainder had no disorder recorded. The median count of disorders per dog during 2013 was one disorder (IQR 0–2, range 0–9).

**Fig. 3:**
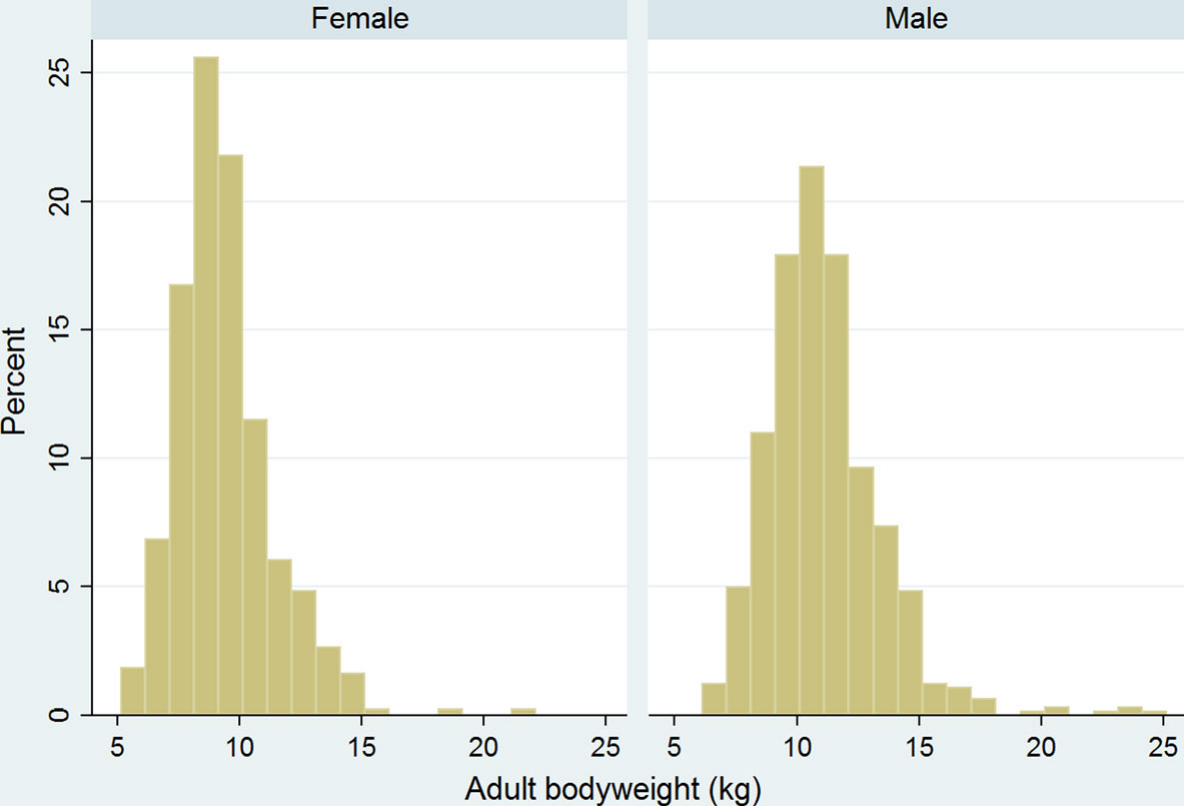
Adult (≥ 9 months age) bodyweight distributions (kg) of 496 female and 665 male Border Terriers under veterinary care during 2013 at 102 clinics in England participating in the VetCompass™ Programme

Of the Border Terriers with information available, 568 (42.8%) were female, 627 (78.7%) were neutered and 453 (55.3%) were insured. The overall median adult bodyweight was 10.1 kg (IQR: 8.8–11.6, range: 5.2–25.0). The median adult bodyweight for males (*n* = 665) (10.9 kg, IQR: 9.6–12.3, range: 6.3–25.0) was higher than for females (*n* = 496) (9.1 kg, IQR: 8.2–10.3, range: 5.2–21.6) (*P* < 0.001) (Fig. 3). The median age of the Border Terriers under veterinary care during 2013 was 5.9 years (IQR: 3.1–9.2 range: 0.3–20.6) years (Table 1).

Of the 66 (2.3%) Border Terriers that died during the study, the median age at death of 12.7 years (IQR 9.3–14.3, range 1.0–17.5). Of the 61 deaths with information recorded, 54 (88.5%) deaths involved euthanasia with the remaining 7 (11.5%) being unassisted.

There were 1832 unique disorder events recorded that encompassed 214 distinct fine-level precision disorders. The most prevalent fine-level disorders recorded were periodontal disease (number of events: 234, prevalence: 17.63%, 95% CI: 15.62–19.79), overweight/obesity (93, 7.01%, 95% CI: 5.69–8.52), otitis externa (89, 6.71%, 95% CI: 5.42–8.19), nails overlong (70, 5.28%, 95% CI: 4.13–6.62), anal sac impaction (63, 4.75%, 95% CI: 3.67–6.03) and vomiting (58, 4.37%, 95% CI: 3.34–5.61) (Table 2). Sex was not associated with the probability of 5 of the top 6 disorders: periodontal disease (*P* = 0.319), overweight/obesity (*P* = 0.408), otitis externa (*P* = 0.364), nails overlong (*P* = 0.316) and vomiting (0.094). Females were associated with anal sac impaction (prevalence in female 6.2% versus in male 3.7%, *P* = 0.036).

**Table 1:** Demography of Border Terriers attending primary-care veterinary practices in England from January 1st, 2013 to December 31st, 2013 (*n* = 1327)

		All records		Complete records only	
Variable	Category	No.	Percent	No.	Percent
Sex	Female	568	42.8	568	42.8
	Male	759	57.2	759	57.2
	Not recorded	0	0.0	~	~
Neuter status	Entire	170	12.8	170	21.3
	Neutered	627	47.3	627	78.7
	Not recorded	530	39.9	~	~
Adult bodyweight (aged ≥9 months) (kg)	5.0–6.9	41	3.1	41	3.5
	7.0–8.9	287	21.6	287	24.7
	9.0–10.9	421	31.7	421	36.3
	11.0–12.9	268	20.2	268	23.1
	13.0–14.9	107	8.1	107	9.2
	≥ 15.0	37	2.8	37	3.2
	Not recorded	166	12.5	~	~
Age category (years)	< 3.0	315	23.7	315	23.8
	3.0–5.9	352	26.5	352	36.6
	6.0–8.9	306	23.1	306	23.1
	9.0–11.9	209	15.8	209	15.8
	≥ 12.0	143	10.8	143	10.8
	Not recorded	2	0.2	~	~
Insurance	Not insured	366	27.6	366	44.7
	Insured	453	34.1	453	55.3
	Not recorded	508	38.3	~	~

**Table 2:** Prevalence of the 25 most common disorders at the greatest diagnostic precision recorded in Border Terriers attending primary-care veterinary practices in England from January 1st, 2013 to December 31st, 2013 (*n* = 1327)

Fine-level Disorder Term	Disorder Count	Prevalence %	95% CI
Periodontal disease	234	17.63	15.62–19.79
Overweight/obesity	93	7.01	5.69–8.52
Otitis externa	89	6.71	5.42–8.19
Nail clip	70	5.28	4.13–6.62
Anal sac impaction	63	4.75	3.67–6.03
Vomiting	58	4.37	3.34–5.61
Conjunctivitis	44	3.32	2.41–4.43
Diarrhoea	43	3.24	2.35–4.34
Heart murmur	34	2.56	1.78–3.56
Lameness	34	2.56	1.78–3.56
Skin mass	33	2.49	1.71–3.47
Pruritus	33	2.49	1.71–3.47
Ear disorder	29	2.19	1.47–3.12
Lethargy	27	2.03	1.35–2.95
Cataract	24	1.81	1.16–2.68
Coughing	24	1.81	1.16–2.68
Seizure disorder	24	1.81	1.16–2.68
Hypersensitivity skin disorder	22	1.66	1.04–2.50
Dog bite injury	21	1.58	0.98–2.41
Epilepsy	21	1.58	0.98–2.41
Degenerative joint disease	21	1.58	0.98–2.41
Dermatitis	19	1.43	0.86–2.23
Gastroenteritis	19	1.43	0.86–2.23
Infectious canine tracheobronchitis (Kennel Cough)	19	1.43	0.86–2.23
Pyoderma	19	1.43	0.86–2.23

There were 49 distinct grouped-level precision disorders recorded. The most prevalent grouped-level precision disorders were dental disorder (*n* = 246, prevalence: 18.54%, 95% CI: 16.48–20.74), enteropathy (155, 11.68%, 95% CI: 10.00–13.53), skin disorder (135, 10.17%, 95% CI: 8.60–11.93), ear disorder (120, 9.04%, 95% CI: 7.55–10.72) and ophthalmological disorder (105, 7.91%, 95% CI: 6.52–9.50) (Table 3).

**Table 3:** Prevalence of the 25 most common disorder groups recorded in Border Terriers attending primary-care veterinary practices in England from January 1st, 2013 to December 31st, 2013 (*n* = 1327)

Grouped-Level Disorder Term	Count	Prevalence	95% CI
Dental disorder	246	18.54	16.48–20.74
Enteropathy	155	11.68	10.00–13.53
Skin disorder	135	10.17	8.60–11.93
Ear disorder	120	9.04	7.55–10.72
Ophthalmological disorder	105	7.91	6.52–9.50
Overweight/obesity	93	7.01	5.69–8.52
Musculoskeletal disorder	90	6.78	5.49–8.27
Claw/nail disorder	81	6.10	4.88–7.53
Anal sac disorder	69	5.20	4.07–6.53
Neoplasia	60	4.52	3.47–5.78
Mass lesion	59	4.45	3.40–5.70
Upper respiratory tract disorder	59	4.45	3.40–5.70
Brain disorder	54	4.07	3.07–5.28
Heart disease	42	3.17	2.29–4.25
Traumatic injury	42	3.17	2.29–4.25
Parasite infestation	36	2.71	1.91–3.74
Endocrine system disorder	35	2.64	1.84–3.65
Lethargy	31	2.34	1.59–3.30
Bite injury	27	2.03	1.35–2.95
Undesirable behaviour	24	1.81	1.16–2.68
Female reproductive system disorder	22	1.66	1.04–2.50
Foreign body	21	1.58	0.98–2.41
Urinary system disorder	18	1.36	0.81–2.14
Congenital disorder	16	1.21	0.69–1.95
Intoxication (poisoning)	14	1.06	0.58–1.76

Syndromic body location classification analysis indicated that 732 (55.16%) Border Terriers had at least one body location affected. The most prevalent body locations affected were the head-and-neck (*n* = 501, prevalence = 37.75%, 95% CI: 35.14–40.43), abdomen (247, 18.61%, 95% CI: 16.55–20.81) and limb (153, 11.53%, 95% CI: 9.86–13.37) (Table 4).

**Table 4:** Prevalence of body locations affected by at least one disorder recorded in Border Terriers attending primary-care veterinary practices in England from January 1st, 2013 to December 31st, 2013 (*n* = 1327)

Body Location	Count	Prevalence	95% CI
Head and neck	501	37.80	35.14–40.43
Abdomen	247	18.61	16.55–20.81
Limb	153	11.53	9.86–13.37
Thorax	100	7.54	6.17–9.09
Anus/Perineum	74	5.58	4.40–6.95
Vertebral column	14	1.06	0.58–1.76
Pelvis	3	0.23	0.08–0.66
Tail	0	0.00	0.00–0.29

Syndromic organ system classification analysis indicated that at least one organ system was affected in 834 (62.85%) Border Terriers. The most prevalent organ systems affected were the digestive (*n* = 425, prevalence: 32.03%, 95% CI: 29.52–34.61), integument (354, 26.68%, 95% CI: 24.31–29.14), connective/soft tissue (148, 11.15%, 95% CI: 9.51–12.97) and auditory (131, 9.87%, 95% CI: 8.32–11.60) (Table 5).

**Table 5:** Prevalence of organ systems affected by at least one disorder recorded in Border Terriers attending primary-care veterinary practices in England from January 1st, 2013 to December 31st, 2013 (*n* = 1327)

Organ system	Count	Prevalence	95% CI
Digestive	425	32.03	29.52–34.61
Integumentary	354	26.68	24.31–29.14
Connective/Soft tissue	148	11.15	9.51–12.97
Auditory	131	9.87	8.32–11.60
Musculoskeletal	113	8.52	7.07–10.15
Ocular	113	8.52	7.07–10.15
Nervous	76	5.73	4.54–7.12
Respiratory	68	5.12	4.00–6.45
Cardiovascular	44	3.32	2.42–4.43
Endocrine	40	3.01	2.16–4.08
Reproductive	38	2.86	2.03–3.91
Urinary	34	2.56	1.78–3.56
Hepatobiliary	13	0.98	0.52–1.67
Haematopoietic	5	0.38	0.16–0.88
Lymphatic	2	0.15	0.04–0.55

Syndromic pathophysiological process classification analysis indicated that at least one affected pathophysiological process was described in 881 (66.39%) Border Terriers. The most prevalent pathophysiologic processes recorded were inflammation (420, 31.65%, 95% CI: 29.15–34.23), nutritional (120, 9.04%, 95% CI: 7.55–10.72), mass/swelling (118, 8.89%, 95% CI: 7.42–10.55), traumatic (106, 7.99%, 95% CI: 6.59–9.58) and infectious (103, 7.76%, 95% CI: 6.38–9.33) (Table 6).

**Table 6:** Prevalence of pathophysiological processes affected by at least one disorder recorded in Border Terriers attending primary-care veterinary practices in England from January 1st, 2013 to December 31st, 2013 (*n* = 1327)

Pathophysiological process	Count	Prevalence	95% CI
Inflammatory	420	31.65	29.15–34.23
Nutritional	120	9.04	7.55–10.72
Mass/Swelling	118	8.89	7.42–10.55
Traumatic	106	7.99	6.59–9.58
Infectious	103	7.76	6.38–9.33
Degenerative	76	5.73	4.54–7.12
Metabolic	57	4.3	3.27–5.53
Neoplastic	50	3.77	2.81–4.94
Parasitic	45	3.39	2.48–4.51
Allergic	41	3.09	2.23–4.17
Behavioural	28	2.11	1.41–3.04
Foreign body-related	25	1.88	1.22–2.77
Hereditary	23	1.73	1.16–2.59
Congenital/Developmental	22	1.66	1.04–2.50
Iatrogenic	22	1.66	1.04–2.50
Intoxicative	14	1.06	0.58–1.76
Immune-mediated	13	0.98	0.52–1.70
Haemostatic	9	0.68	0.36–1.28
Thermoregulatory	1	0.08	0.01–0.43
Effusion	0	0.00	0.00–0.29

## Discussion

This study represents the largest analysis of breed health in Border Terriers based on primary-care veterinary records to date and provides data on the demography and common disorders of the general population of Border Terriers in England. The syndromic analyses provide additional perspectives on the relative predilection of body locations, body systems and pathophysiologies to disorders that can assist a more holistic interpretation of the health of the Border Terrier. A paradoxical trend towards decreasing ownership in recent years was identified despite some opinion that the breed is generally healthy. The most common disorders identified in Border Terriers were periodontal disease, overweight/obesity and otitis externa. These results can provide an evidence base to support development of health priorities in Border Terriers that can contribute positively to reforms to improve health and welfare within the breed.

Application of demographic techniques can improve the reliability of interpretation and generalisation from canine health studies results [21–23]. For example, there is now substantial evidence to suggest that insurance uptake has a strong effect on diagnostic probabilities across disorders in dogs (i.e. what proportion of the true cases are actually diagnosed). Compared with non-insured dogs, insured dogs have 4.0 times the odds of diagnosis with hyperadrenocorticism [24], 4.0 times the odds of diagnosis with cruciate disease [25], 3.6 times the odds of diagnosis with mitral valve disease [26], 2.6 times the odds of diagnosis with chronic kidney disease [27], 1.9 times the odds of diagnosis with patellar luxation [28] and 1.6 times the odds of diagnosis with corneal ulceration [7]. The impact of insurance on diagnostic probability appears to increase as conditions become more expensive or complex to diagnose. Access to pet insurance may lower financial barriers to diagnosis for owner and veterinarians and consequently encourage earlier and more frequent veterinary visits as well as facilitating greater diagnostic freedom [29]. Owners who are motivated to purchase pet insurance may also have stronger emotional bonds with their pets or a higher commitment to providing the best medical therapy [30]. The current study identifies a relatively high uptake of pet insurance for the Border Terrier with 55.3% of dogs insured where this information was recorded. This may promote higher diagnostic rates and should be considered when comparing results with other breeds that may have differing insurance uptakes.

The median longevity of Border Terriers in the current study was 12.7 years which is longer than the median longevity of 12.0 years reported across all dogs in England [31]. Factors associated with increasing longevity in dogs include decreasing bodyweight, being crossbred, being female and specific breed types [31–34]. However, the spectrum of true influences on longevity are likely to be much more complicated and to include a wide range of genetic, epigenetic and environmental effects with each having non-linear direct and interactional effects [35–37]. Consequently, extrapolating longevity within a breed to act as a proxy measure of health in the breed is fraught with difficulties. However, the current study suggests that the Border Terrier is a relatively long-lived breed and, as such, the longevity results do not provide specific evidence that poor health is limiting the lifespan of the breed any more than for dogs in general.

Awareness of breed-related predispositions to specific diseases has important implications towards a better understanding of the aetiology and prevention of these diseases, especially in relation to the genetic component of causality. The modern dog has a unique population structure with each breed emanating from a relatively closed pool of homologous parent breeds that themselves arose from a limited number of founders [38, 39]. This means that there is reduced genetic diversity within breeds and greater genetic divergence between breeds [38, 40, 41]. Consequently, awareness of strong breed predisposition to individual disorders facilitates genetic linkage or association studies on relatively small populations of dogs that can help to unravel the genetics of complex disorders [42, 43]. Knowledge of key breed predispositions can assist breeders to select against lines or conformations that promote the predisposition and facilitate more timely diagnosis and clinical management once the condition occurs [44, 45]. It is also likely that breeds with shared ancestry may also share disease proclivities [46]. The Border Terrier shares common ancestry with the Dandie Dinmont Terrier and the Beddlington Terrier and future breed health profiles may be expected to show similarities in common disorders between these three breeds [47].

However, there is also a real risk that certain breeds can become defined by heightened awareness of predisposition to particular disorders that may not necessarily be either common or severe, regardless of the relative risk of occurrence in that breed. A textbook dedicated to the *Breed Predispositions to Disease in Dogs and Cats* reports the Border Terrier as predisposed to 6 disorders: generalised sebaceous gland hyperplasia, canine epileptoid cramping syndrome, epilepsy, corneal ulceration, ectopic ureter and dystocia [48]. In the case of the Border Terrier, the results of the current study suggest that epilepsy is the only one of the 6 reported breed predispositions that appears among the 25 most commonly diagnosed disorders (Table 2). This highlights the importance of the distinction between predisposition (relative risk) and prevalence (absolute risk) and of understanding the different stories told by these two metrics [49].

Periodontal disease is a collective term for a spectrum of pathologies affecting periodontal tissues such as the gingiva, periodontal ligament and alveolar bone [50]. Periodontal disease is reversible initially when just the gingiva are inflamed but the disease becomes irreversible as the supporting structures of the tooth are progressively damaged [51]. Many of the oral pathologies intrinsic to periodontal disease may be painful for affected dogs. Dentoalveolar and gingival pain, whether chronic or acute, is well documented in the human population [52, 53] and negative quality of life impacts have been reported for human [54, 55] and animal species [50, 56]. Periodontal disease has been linked with systemic diseases in dogs including chronic kidney disease [57, 58] and endocarditis [59] as well as histopathologic changes in kidney, myocardium and liver [60]. Consequently, irreversibility, pain and systemic effects mark out periodontal disease as an important disease in dogs. Dental disease was the most common disorder recorded in Border Terriers in the current study, with 17.63% dogs recorded with periodontal disease which was more than twice the prevalence of the next highest disorder. The current study included only dental disorder cases where at least one clinical management intervention such as dentistry or dietary change was recommended. This approach has been used in previous studies [15, 61, 62] and aimed to preferentially include cases that were considered to be more serious by the attending veterinarian. Periodontal disease has previously been reported as the second most common diagnosis in dogs in England, with 9.3% of dogs affected in a study that used a similar methodology to the current study [15]. Similarly, aspects of dental disease including dental calculus (20.5%) and gingivitis (19.5%) were the most common recorded disorders among US veterinary consultations although this study reported all cases regardless of perceived severity [63]. The high absolute and relative prevalence for periodontal disease reported in the current study suggest that the Border Terrier should be considered as a predisposed breed for this condition and also that periodontal disease should be considered a priority condition for the breed. Certainly, greater emphasis on oral hygiene and prevention of dental disease could be targeted to owners of both affected and unaffected Border Terriers in order to prevent undesirable sequelae.

Seizure disorder (1.81% prevalence) and epilepsy (1.58% prevalence) were ranked at 17th and 20th position respectively among the most common disorders recorded in the current study. Although these terms may describe the same underlying pathologies, they were reported separately in this study because the study was restricted to reporting diagnostic terms as recorded in the clinical records. Despite apparently low ranking placements (17th and 20th), these conditions are marked out as significant by their absence from the list of most common disorders in dogs overall in England that used a similar methodology [15] which suggests a true predisposition for neurological disease may exist in the Border Terrier. A study of epilepsy of unknown origin reported an odds ratio of 2.70 for Border Terrier compared with crossbred dogs and singled out the Border Terrier as the most predisposed breed in the study [6]. However, diagnostic reporting for seizure disorders in the Border Terrier is complicated by reports of canine epileptoid cramping syndrome (CECS) in the breed. Also known as Spike’s Disease, CECS has been recognised for over a decade in the Border Terrier but remains poorly clinically characterised to date [5]. It is one of a group of movement disorders called paroxysmal dyskinesias that present as involuntary muscular movements that can occur suddenly in dogs that have normal motor function and that show no evident neurological deficits between episodes [64]. Paroxysmal dyskinesias could be confused clinically with simple focal seizures but can be distinguished by typical features of the dyskinesia episodes which include full consciousness throughout, the types of movements exhibited, duration of each episode (focal seizures often last under 10 min whereas episodes of CECS may extend beyond 2 h), the absence of typical autonomic signs often observed with seizures such as urination, defecation and hypersalivation, and the lack of characteristic postictal behaviour [5, 64, 65]. Additionally, at a more advanced diagnostic level, CECS will rarely show seizure activity on ictal EEG recordings and are not effectively controlled by conventional antiepileptic medications [65]. However, limited awareness of CECS by veterinarians and owners combined with the similarities between CECS and focal seizures suggest that some cases of CECS may be still misclassified as poorly responsive seizure disorders [5]. Enhanced vigilance for CECS in the Border Terrier may result in more reliable diagnosis and management of this condition.

Overweight/obesity was the second most common disorder recorded in the current study, affecting 7.01% of Border Terriers. This value is similar to the 6% prevalence recorded in dogs overall in England in a study using a similar methodology [15]. This suggests that the Border Terrier is not especially predisposed to obesity compared with other breeds but it also highlights the condition as common in the breed and therefore still warranting substantial clinical concern. It is worth noting that this reported prevalence value for overweight/obesity may substantially under-estimate the true prevalence because retrospective studies using secondary clinical data such as the current study have consistently been shown to under-report obesity compared with prospective studies that have reported prevalence values from 25% to 41% [66–68]. Despite lacking a specific breed predisposition, obesity is still clinically relevant to Border Terriers because of association with disorders including diabetes mellitus, cardiovascular, skin and musculoskeletal disease, exercise and heat intolerance, metabolic syndrome and increased surgical and anaesthetic risk [68–71]. Consequently, prevention and reduction of overweight/obesity should be considered a health priority in Border Terriers because of the high prevalence, associated health problems and reversible nature of the disorder [72].

This study accepted the benefits and disadvantages from the application of secondary veterinary clinical data for research [73]. Limitations from the use of such data for research have been reported previously [7, 15, 61, 62, 73]. Studies based on clinical records may under-estimate the true disease burden by inclusion of more severely affected individuals that present for veterinary care [74]. The practices included in the current study were situated mainly in central and south-east England and therefore may not be fully representative of the overall veterinary practice structure in England. Case definitions and diagnosis recording relied heavily on the clinical acumen and note-making of attending practitioners [15]. The current study ranked disorders based on prevalence but additional data on duration and severity are also required for effective welfare prioritisation of disorder [75, 76].

## Conclusions

This study of over one thousand Border Terriers documented a trend towards reducing ownership in England. The Border Terrier is shown to be a relatively long-lived breed. The most common disorders recorded were periodontal disease, overweight/obesity and otitis externa. Predisposition to periodontal disease and epilepsy is suggested. These results can provide a comprehensive and useful evidence resource to support breed-based health plans for the Border Terrier that can contribute positively to reforms to improve health and welfare within the breed.

## Abbreviations

CECS: canine epileptoid cramping syndrome
CI: confidence interval
EPR: electronic patient record
GISID: Generic Illness Severity Index for Dogs
IQR: interquartile range
OR: odds ratio
